# Brain Midline Shift Measurement and Its Automation: A Review of Techniques and Algorithms

**DOI:** 10.1155/2018/4303161

**Published:** 2018-04-12

**Authors:** Chun-Chih Liao, Ya-Fang Chen, Furen Xiao

**Affiliations:** ^1^Institute of Biomedical Engineering, National Taiwan University, No. 1, Sec. 1, Renai Rd., Taipei City 10051, Taiwan; ^2^Department of Neurosurgery, Taipei Hospital, Ministry of Health and Welfare, No. 127, Siyuan Rd., New Taipei City 24213, Taiwan; ^3^Department of Medical Imaging, National Taiwan University Hospital, No. 7, Zhongshan S. Rd., Taipei City 10002, Taiwan; ^4^Department of Neurosurgery, National Taiwan University Hospital, No. 7, Zhongshan S. Rd., Taipei City 10002, Taiwan

## Abstract

Midline shift (MLS) of the brain is an important feature that can be measured using various imaging modalities including X-ray, ultrasound, computed tomography, and magnetic resonance imaging. Shift of midline intracranial structures helps diagnosing intracranial lesions, especially traumatic brain injury, stroke, brain tumor, and abscess. Being a sign of increased intracranial pressure, MLS is also an indicator of reduced brain perfusion caused by an intracranial mass or mass effect. We review studies that used the MLS to predict outcomes of patients with intracranial mass. In some studies, the MLS was also correlated to clinical features. Automated MLS measurement algorithms have significant potentials for assisting human experts in evaluating brain images. In symmetry-based algorithms, the deformed midline is detected and its distance from the ideal midline taken as the MLS. In landmark-based ones, MLS was measured following identification of specific anatomical landmarks. To validate these algorithms, measurements using these algorithms were compared to MLS measurements made by human experts. In addition to measuring the MLS on a given imaging study, there were newer applications of MLS that included comparing multiple MLS measurement before and after treatment and developing additional features to indicate mass effect. Suggestions for future research are provided.

## 1. Introduction

### 1.1. History of Midline Shift as an Imaging Feature

Human head is roughly bilaterally symmetric. Although there are functional differences between hemispheres of the brain, the gross morphology follows the rule [1]. Both cerebrum and cerebellum are symmetric with lobes, ventricles, and deep nuclei of similar size and shape in both hemispheres. Subtle structural asymmetry plays no role in clinical diagnostic neuroradiology [2]. From pathological examinations, physicians have already known that intracranial mass can cause brain shift, followed by herniation, brainstem compression, and death. Therefore, they rely on shift of midline structures for aiding diagnosis from the very beginning of neuroimaging. Shift of calcified pineal gland on plain X-ray was used initially, followed by pneumoencephalography and angiogram [3].

**Figure 1:**
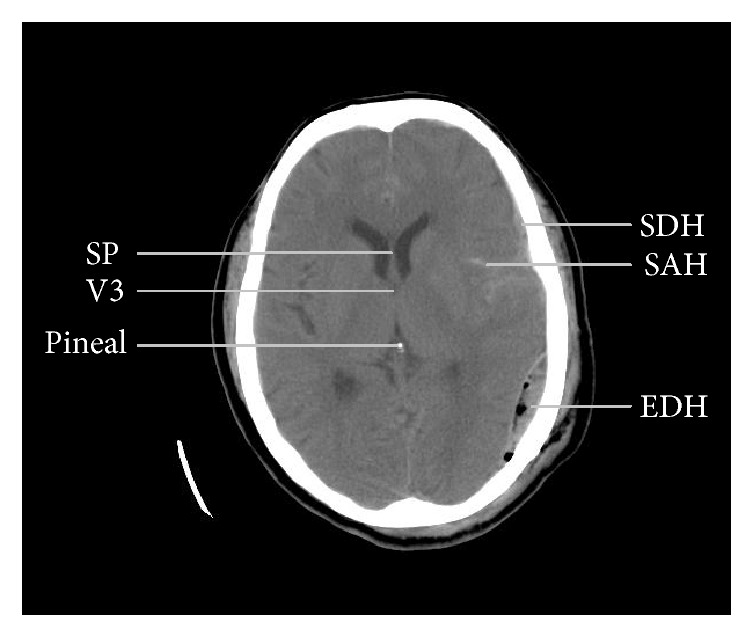
A computed tomographic image from a patient with traumatic brain injury showing anatomical landmarks used to measure midline shift (2 mm in this image) and different types of intracranial hemorrhage. SP: septum pellucidum, V3: third ventricle (only the most rostral part shown), SDH: subdural hematoma, SAH: subarachnoid hemorrhage, and EDH: epidural hematoma.

After the invention of ultrasound (US), computed tomography (CT), and magnetic resonance imaging (MRI), cross-sectional imaging becomes possible with greatly improved resolution and tissue contrast [2, 3]. While the cerebrospinal fluid- (CSF-) containing third ventricle (V3, Figure 1) is more easily identified on US images [4], most authors describe the degree of displacement of the septum pellucidum (SP, Figure 1), a thin membrane between the frontal horns (FHs) of the lateral ventricles, relative to the ideal midline (iML) on CT images [5, 6]. Whether the pineal gland, the V3, or the SP is used, deviation of the given midline structure from the iML is termed midline shift (MLS). Since symmetry plays a key role in radiologic evaluation of the brain, any shift of midline structures is presumed to represent a mass lesion on the side from which the midline is displaced [2]. For practical purposes, there are no acute “sucking” brain lesions that draw the midline toward themselves.

### 1.2. Using Midline Shift as a Quantitative Indicator of Mass Effect to Predict Outcome in Trauma Patients

As early as 1783 Alexander Monro deduced that the cranium is a “rigid box” filled with a “nearly incompressible brain” and that its total volume tends to remain constant [8]. The doctrine states that any increase in the volume of the cranial contents (e.g., brain, blood, or CSF) will elevate intracranial pressure (ICP). Furthermore, if one of these three elements increases in volume, it must occur at the expense of volume of the other two elements. In 1824 Kellie confirmed many of Monro's early observations [9]. According to this doctrine, focal intracranial pathology can damage all intracranial structures by reducing their perfusion from increased ICP if all compensatory mechanisms are exhausted. Such phenomenon is called “mass effect.”

In NIH Traumatic Coma Data Bank, a large prospective multicenter study, the authors have examined data derived from the initial CT scans of 753 patients with severe traumatic head injury (TBI), defined as a Glasgow Coma Scale (GCS) score of 8 or less [36]. When the CT findings were related to increased ICP and death, the most important characteristics of the scans were MLS, compression or obliteration of the perimesencephalic cisterns, and the presence of subarachnoid blood (subarachnoid hemorrhage, SAH) [10]. In many subsequent studies, presence of MLS was related to increased ICP and worse prognosis [11–17]; however interaction with the presence of intracranial lesions and other CT parameters exists, as summarized in a previous review [5]. MLS on CT continues to be a noninvasive estimator of ICP in TBI patients before actually measuring it during surgery and is regarded as an imaging feature supporting Monro-Kellie doctrine. A dose-dependent relationship between MLS and outcome of TBI patients has been demonstrated [18]. Similar relationship also exists between MLS and consciousness in patients with acute hemispheric mass [37].

Although the classification schemes were highly variable in previous reports, MLS is a quantitative measurement that can be done on unenhanced or contrast-enhanced images. It can have positive and negative values and can be defined as 0 in a subject with no shift at all. As the MLS can be measured in every brain, with or without pathology, it has become an integral part in evaluating brain images. However, MLS is less suitable for representing mass effect when there are multiple lesions [5]. On the other hand, perimesencephalic cisternal compression is able to reveal mass effect in the presence of bilateral, multiple, or posterior fossa lesions; but it is at best considered semiquantitative measurement.

### 1.3. Standardization of Midline Shift Measurement

To further decrease variations in MLS measurement in TBI patients, Brain Trauma Foundation (BTF) proposed a standardized protocol of the CT imaging procedure in 2006. Standardized methods of hematoma volume estimation using the “*ABC*” method and MLS measurement were proposed [6, 38]. They suggested using 5-mm axial (horizontal) slices from the foramen magnum to the sella and 10-mm slices above the sella, parallel to the orbitomeatal line [6]. As newer CT scanners are able to obtain isotropic voxels allowing image reconstruction in any anatomic plane without loss of resolution, many hospitals now use 5-mm slices throughout the procedure [2].

**Figure 2:**
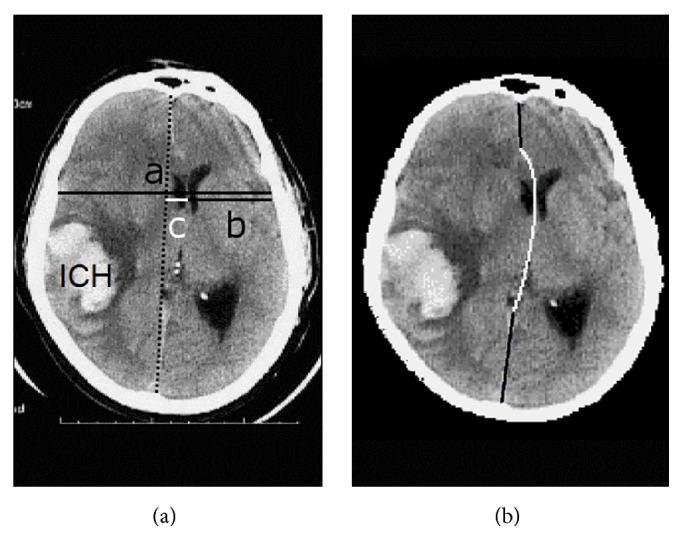
Assessment of midline shift (MLS) on an image of intracerebral hematoma (ICH) compressing the brain. (a) Although determination of the MLS by first measuring the width of the intracranial space (*c* = *a*/2 − *b*) was suggested by the guideline, many neurosurgeons measured it by first drawing the ideal midline (dotted line). (b) Our computational model for the deformed midline included a quadratic Bezier curve (white) between two line segments (black). Adapted from [7].

On a given axial image, the MLS is measured at the level of the foramen of Monro (FM), which is the channel connecting the FHs of the lateral ventricles to the V3, as shown in Figures 1 and 2. At the level of the FM, only the most superior part of the V3 can be seen, as illustrated in Figure 2. The largest anterior-posterior diameter of the V3 is usually caudal to this level [4]. The BTF guideline suggested determining the MLS (“*c*” in Figure 2(a)) by first measuring the width of the intracranial space (“*a*”), followed by measuring the distance from the bone to the SP (“*b*”). Then the MLS can be determined by calculating MLS = (*a*/2) − *b*. In the guidelines, BTF also recommended emergency surgery for any traumatic epidural (EDH), subdural (SDH), or intracerebral hematoma (ICH) causing an MLS larger than 5 mm [39–41].

Because the skull is not always symmetric and the patient may not be perfectly aligned during CT examination, many specialists measure MLS by first drawing the iML joining the most anterior and posterior visible points on the falx (dotted line in Figure 2(a)) and then measuring the farthest point on the SP (the rightmost point of the white horizontal line segment in Figure 2(a)) as perpendicular from the iML. Such method has also been shown to have high interobserver agreement in patients with spontaneous ICH [42]. Moreover, determining the iML is easier than determining the width of the intracranial space when the skull is deformed or removed by surgery or trauma.

After proving its prognostic value in TBI patients, MLS is widely used in the assessment of neurological diseases as an indicator of mass effect. Since every disease has its own natural history, measurement and analysis of the MLS should be performed under the context of the primary diagnosis, as tabulated in Table 1. In this paper, we review commonly used imaging methods for MLS measurement and their applications to different diseases in Section 2. In Section 3, we review algorithms for automated MLS measurement and their advantages and limitations. Newer applications including measurements of MLS on posttreatment images and development of novel features of mass effect are reviewed in Section 4 and finally concluding remarks are provided.

## 2. Methods

### 2.1. Computed Tomography

CT uses a computer to reconstruct cross-sectional images from measurements of X-ray transmission through thin slices of patient tissue [2]. Noncontrast CT scan is the imaging modality of choice for TBI because of widespread availability, rapid imaging acquisition, superior bone detail, capability of whole-body imaging in multiply injured patients, low associated costs, and compatibility with most medical devices allowing examination of unstable patients [6]. On CT images, it is possible to measure the MLS using the SP, the pineal gland, or the V3 as an anatomical landmark.

Generally, brain CT is performed for acute neurological conditions and MRI for subacute or chronic cases. In addition to TBI, stroke is another important acute neurological condition requiring brain imaging. National Institutes of Health Stroke Scale (NIHSS) score is often used to quantify the neurological impairment. Noncontrast CT scan is the preferred initial imaging study for stroke patients because it can identify hyperdense hemorrhage and differentiate it from cerebral infarction, guiding immediate intervention along with NIHSS score. However, early signs of infarctions on CT are subtle and precise identification of the infarcted area is usually impossible [2].

The emergence of brain swelling is the most feared life-threatening consequence of a large-territory ischemic stroke. The term malignant middle cerebral artery (MCA) infarction, introduced in 1996, was originally defined as infarction of the entire MCA territory, or even larger areas, appearing as areas of decreased attenuation (hypodensities) on CT within 48 hours [43]. Neurological deterioration usually occurs in most patients within 72 to 96 hours, but some patients may experience deterioration over the next few days [44]. CT is also the modality of choice for unstable patients with MCA infarcts with swelling requiring follow-up imaging. The degree of the MLS is commonly used as the benchmark for radiographic deterioration. However, definition varies among studies [44]. Once malignant MCA infarction is diagnosed, decompressive craniectomy (DC) with expansive duroplasty is the only effective treatment. DC is also commonly performed alone or in conjunction with hematoma removal on patients with increased ICP after TBI [45].

Pullicino et al. measured several parameters on axial CT performed within 48 hours of onset in 118 consecutive patients with severe acute hemispheric stroke [19]. Crude risk factors for 14-day mortality, which occurred in 46 patients, were a lesion volume of 400 ml or larger, an SP MLS of 9 mm or larger, a pineal MLS of 4 mm or larger, intraventricular hemorrhage, and coma on admission. Only the SP MLS was significantly correlated to survival in multivariate analysis but the two MLS measurements were highly correlated with a correlation coefficient of 0.82.

Lam et al. analyzed features on axial CT performed within 24 hours of symptom onset in 55 patients with acute extensive MCA infarct [20]. The authors categorized their MLS measurement into 3 groups: no MLS, MLS smaller than 10 mm, and MLS larger than 10 mm. They also did not describe which landmark was used to measure the MLS. Single explanatory variable analysis showed NIHSS, presence of MLS, MLS larger than 10 mm, extent of infarct, presence of hydrocephalus, effacement of subarachnoid space or cella media, and loss of corticomedullary differentiation were associated with the 30-day mortality (14 patients). Logistic regression analysis showed that the extent of infarct and NIHSS were the only independent predictors. Because brain edema usually develops later, the authors considered “early” MLS on the first day a highly specific but insensitive sign.

Park et al. used diffusion-weighted MRI (DWI) within 14 hours and CT 24 ± 4 hours after stroke onset in 61 patients to assess the infarct volume and MLS at SP [21]. The degree of brain atrophy was also evaluated using the bicaudate ratio. For the patients who presented with an acute hemispheric infarction, an infarct volume larger than 220 ml or MLS larger than 3.7 mm on the follow-up CT approximately 24 h after stroke onset predicts malignant infarction, which was noted in 21 patients. For infarction patients with less atrophic brains, defined by a bicaudate ratio of less than 0.16, an initial infarct volume larger than 160 ml in a DWI within 14 h after stroke onset is highly predictive of a malignant course.

Spontaneous ICH is the most common subtype of hemorrhagic stroke. The decision about whether and when to surgically remove ICH usually depends on hematoma volume and location [46]. Similar to traumatic hematoma, the volume of spontaneous ICH is estimated using the ABC formula [22, 23, 38]. The MLS measured at the SP or the pineal gland is also used to quantify the progression of mass effect after ICH. Zazulia et al. found 17 instances of MLS progression, defined as an increase of more than 2 mm, in 76 patients having repeated CT scans after spontaneous supratentorial ICH [22]. Among them, 10 occurred within 2 days and were associated with hematoma enlargement, and 7 occurred later and were associated with edema progression. Progression of mass effect due to edema occurred with larger hemorrhage volumes. Compared to the pineal MLS, the SP MLS was a more sensitive measurement. However, the clinical significance of late-onset edema and patient outcome were not reported.

Song et al. correlated coma (GCS score of 8 or less) and anisocoria with CT findings in 118 patients with spontaneous supratentorial ICH [23]. Univariate analysis revealed that hematoma volume, the score of intraventricular hemorrhage, and the amplitude of the MLS were related to coma and anisocoria. The mean MLS were 1.3, 5.9, and 10.1 mm in patients without coma, those with coma but not anisocoria, and those with both coma and anisocoria, respectively. The authors did not mention whether any specified landmark was used to measure the MLS. The 30-day mortality was 33.9% and whether any patient had surgery was not reported. In addition, their clinical findings were not correlated to outcome.

Chronic subdural hematoma (cSDH) is composed of thick black liquid like motor oil containing lysed blood clot. It is usually encountered in the elderly and evolution from acute SDH to cSDH takes several weeks [2, 47]. Clinical symptoms and signs of cSDH are less dramatic than those of acute SDH, which is rapidly lethal if left untreated. On CT images, cSDH appears as low-attenuation collection outside the brain. The MLS can be significant, especially in patients with atrophic brains. Clinically, most patients with cSDH present with headache or mild limb weakness (hemiparesis) even with large MLS. Bilateral cSDH is common. When it occurs, the midline is pushed back into its normal position, making the MLS less useful in such patients. Other imaging features must be added to adequately evaluate the mass effect.

Instead of mortality, MLS is correlated to other variables in patients with cSDH. Jukovic and Stojanovic evaluated 83 patients with 53 unilateral and 30 bilateral cSDHs to determine the MLS threshold for hemiparesis [24]. The authors did not describe how they measured the MLS. Their results suggested that in unilateral cSDH the threshold of MLS could be at 10 mm; for bilateral cSDH the threshold was 4.5 mm. Interestingly, patients with unilateral cSDH are more likely to have both hemiparesis (44 patients) and MLS (48 patients), but the receiver operating characteristic curve was smaller than that derived from bilateral cSDH patients. The authors did not report how their patients were treated but did find hemiparesis contralateral to the side of the thicker hematoma layer in bilateral cSDHs. Some of their patients might have asymmetrically distributed “bilateral” lesions which behave like unilateral cSDH clinically and radiologically.

In some patients with cSDH, the consciousness is impaired. Sucu et al. evaluated 45 patients with cSDH who underwent burr-hole or twist-drill craniostomy [25]. They compared level of consciousness of patients measured by GCS score, MLS at the pineal gland, and the SP both in the preoperative and early postoperative period. In every patient, the pineal MLS was almost always smaller than the SP MLS on both pre- and postoperative CT images. The postoperative CT scans were evaluated just after removal of drainage catheters, 2 to 4 days postoperatively. Of 45 patients included, 28 had impaired consciousness defined by GCS score less than 15. Half of them had GCS scores of 13 (8 patients) and 14 (6 patients). In patients with cSDH and impaired consciousness, they found that the likelihood of GCS returning to 15 after operation was increased if the SP MLS was 10 mm or greater. The authors concluded that cSDH evacuation is unlikely to restore consciousness if the associated MLS is not large enough to explain a poor level of consciousness. In other words, a small MLS makes it more likely that there is a separate cause. In both studies on cSDH, the MLS thresholds are considerably larger than those used in TBI or MCA infarction patients. Such differences can be explained by different pathophysiology and higher degree of brain atrophy in cSDH patients.

Brain abscess is defined as a focal suppurative process within the brain parenchyma. In earlier stages of brain abscess called cerebritis, the suppurative lesion is poorly demarcated from surrounding brain. When the abscess capsule forms in later stages, contrast-enhanced CT and MRI scans show a well-defined, usually smooth and thin, rim of enhancement (ring enhancement) [2, 47]. Demir et al. evaluated CT and MRI images of 96 patients with clinical diagnoses of brain abscesses retrospectively [26]. They collected imaging features in terms of the number and location and size of lesions and the presence and extent of perilesional edema and the MLS. An imaging severity index was constructed accordingly. In these patients, 86 underwent surgery, mostly aspiration (72 patients). The authors probably measured the MLS near the SP or the V3, as shown in their figures, but details were not provided. They classified MLS as mild (smaller than 5 mm), moderate (between 5 and 10 mm), or severe (larger than 10 mm) and then added up scores obtained from other parameters. They showed a negative correlation between imaging severity index and initial GCS. There was a significant difference between the clinical and imaging parameters of patients with an adverse event compared with patients with good recovery.

After DC for TBI or malignant MCA infarction, patients have large skull defects. They undergo cranioplasty after brain edema subsided for protection and cosmesis. In addition to determining whether DC is required, MLS was also used to predict neurological improvement after cranioplasty. Lin et al. enrolled 56 cranioplasty patients, 35 with MLS ranging from 1 to 12 mm and 21 without MLS, and analyzed their clinical characteristics. Forty-six of their patients had DC for TBI or spontaneous ICH and 10 for large infarction or intracranial infection [27]. All of them had undergone large unilateral DC with skull defect diameters larger than 100 mm. There were significant improvements in GCS, arm muscle power, and leg muscle power scores one year after cranioplasty. A significant larger improvement in the GCS score was observed in the MLS group. Eight patients in the MLS group had sunken brain, which implies larger antecedent lesion caused by TBI or stroke. Large brain insults are frequently related to syndrome of the trephined (ST) after DC when brain edema resolves with time. The authors attribute neurological improvement to resolution of ST, but they did not report how many of the 9 patients with MLS and GCS score improvement had sunken brain.

### 2.2. Magnetic Resonance Imaging

MRI is a technique that produces tomographic images by means of magnetic fields and radio waves [2]. It provides outstanding soft tissue contrast, substantially better than any other imaging modality including CT and US. In any patient in whom intracranial neoplasm or infection is a consideration, contrast-enhanced MRI is the preferred study as these lesions can be identified as abnormal enhancement. Because the MRI signal is very weak, prolonged imaging time and patient cooperation are often required, making it less suitable for examining unstable patients. Reconstructed using standard orthogonal planes, namely, axial, sagittal, and coronal, the axial MRI images angle slightly differently from their CT counterparts, which are reconstructed parallel to the orbitomeatal line. Despite such difference, measuring the MLS on MRI images and on CT images is essentially the same process. Once the slice containing relevant anatomical landmark is selected, MLS can be determined by measuring the distance between that structure and the iML, or half the width of the intracranial space, as described in Section 1.3.

Compared to CT, MRI DWI detects the infarcted volume within the first few hours, allowing early identification of the involved territory and prediction of brain swelling, including malignant MCA infarction. However, CT remains mainstay in diagnosing brain swelling on follow-up imaging when clinical worsening occurs. In a prospective, multicenter, observational cohort study, Thomalla et al. studied patients with acute MCA infarction using MRI techniques including DWI, perfusion imaging, and MR-angiography within 6 hours of symptom onset [28]. Of 140 patients included, 27 developed malignant MCA infarction, defined as NIHSS score worsening and large MCA infarction on follow-up MRI or CT of at least two-thirds of the its territory with compression of ventricles or MLS. In this study, MLS is used as an end point rather than an outcome predictor. Once it is detected along with large infarction on MRI or CT, malignant MCA infarction can be diagnosed. However, a quantitative definition of MLS was not given. Although CT is the safest examination for unstable patients with neurological deterioration, some patients could have MLS detected on follow-up MRI prior to clinical worsening. The prespecified threshold of a DWI lesion volume larger than 82 ml predicted malignant infection with high specificity but sensitivity was low. The authors concluded that, in a subset of patients with small initial DWI lesion volumes, repeated diagnostic tests are required. For the same reason, routine CT follow-up with MLS measurement was also performed by Park et al. as described previously in Section 2.1 [21].

Cerebral venous thrombosis (CVT) is a rare stroke subtype with a highly variable clinical course. Yii et al. conducted a retrospective study of 106 consecutive patients with imaging-confirmed CVT from 1997 to 2010 [29]. Their study showed that venous infarcts and hyperintensity on DWI were associated with clinical deterioration. Other imaging features, including parenchymal hemorrhage, vasogenic edema, MLS, and thrombosis location, were not predictive of clinical deterioration. These results indicated that CVT has a different natural history from MCA infarction.

Intracranial neoplasm and abscess can have similar subacute history and focal neurological deficit. Both abscess and tumor have perifocal (surrounding) edema, but the former tends to have ring enhancement on CT and MRI images while the latter can be solid or cystic with thick, irregular wall. Demir et al. did contrast-enhanced MRI on patients with clinical diagnoses of brain abscess when there was no contraindication [26]. On MRI, MLS can be measured using the same technique as on CT. These results can be directly compared and collected together for further statistical analysis, as described in Section 2.1.

Baris et al. reviewed the MRI images of 40 patients with primary and 40 with metastatic intra-axial supratentorial brain tumors [30]. Supratentorial primary solitary brain tumor group was also subdivided as glioblastoma multiforme (GBM) subgroup (24 patients) and other-than-GBM subgroup (16 patients). MLS, tumor volume, perifocal edema volume, and the ratio of edema to tumor were measured. The pathological diagnoses of primary tumors other than GBM include tumors of lower grade, less aggressive subtype. The authors used axial FLAIR images to measure subfalcine herniation, which seemed to be synonymous with MLS. However, they did not report whether any specific landmark, such as the SP, was used. The degree of MLS was categorized as grade 1 herniation when MLS was smaller than 5 mm and as grade 2 herniation when MLS was larger. Their results showed that MLS and tumor volume of the primary tumor group were greater than metastasis group while the edema volume relative to tumor volume was less. MLS larger than 5 mm was more common in primary tumors. Since larger tumors have larger MLS and smaller additional space for edema, tumor size difference between groups may contribute to these differences.

Compared to malignant tumors, benign brain tumors have different biological behavior and natural history. Zeidman et al. reviewed 21 who had serial MRI brain scans to determine the growth rate of nonoperated meningiomas [31]. The decision not to have surgery included absence of related neurologic symptoms or signs and concern about high operative risk of neurologic impairment. They concluded that mean volumetric growth rate was significantly greater than the planimetric growth rate. While they also recorded special imaging characteristics including calcification, T2 hypointensity, dural tail, mass effect, and MLS, none of them were correlated to the growth rate. As meningiomas are mostly benign slow-growing tumors, the ICP remains normal until the tumor becomes very large. Therefore, MLS plays little role in following meningioma patients.

### 2.3. Ultrasound

US imaging is performed by using the pulse-echo technique. The US transducer converts electrical energy into a brief high-frequency sound pulse that is transmitted into patient tissues, and then it becomes a receiver, detecting echoes of reflected sound energy [2]. Instead of imaging the whole anatomical volume and reconstructing standardized axial, sagittal, and coronal slices, US images are produced in any anatomic plane by adjusting the orientation and angulation of the transducer and the position of the patient. Visualization of anatomical structures by US is limited by bone and by gas-containing structures such as the skull and the bowels.

Except in infants, US is not the first-line diagnostic tool for brain imaging. Patients with neurological conditions first undergo CT or MRI examination. Then, US can be used to evaluate the carotids or to evaluate the intracranial vessels with transcranial color Doppler sonography (TCCS) techniques. An important advantage of US is the convenience for bedside examination, which is helpful to unstable patients who may have ventilators, monitors, and intravenous pumps, making transportation both cumbersome and risky [4, 32–35].

Seidel et al. performed bedside TCCS examination to study MCA flow patterns in stroke patients [48]. They concluded that TCCS can provide rapid and reliable data regarding stroke subtype and mechanism immediately after onset, but the examination could not be performed due to insufficient temporal acoustic window in 17 of their 84 patients. In addition, they also pioneered MLS measurement by using US, aided by TCCS [33]. After identifying the arteries of the circle of Willis, the depth of the insonation window was adjusted so that the midbrain in the center of the image and contralateral skull became visible. From this position, the transducer was tilted upward by 10 degrees to identify the V3 using its hyperechoic margins and the surrounding hypoechogenic thalamus and hyperechoic pineal gland. Though somewhat tilted, the US scanning plane is approximately horizontal. The distances between the US probe and the center of the V3 were measured from both sides of the head. These two distances, *d*1 and *d*2, can then be used to calculate MLS according to the formula MLS = (*d*1 − *d*2)/2. Mathematically, this formula is the same as the MLS formula described in Section 1.3.

In brains with degenerative diseases, it is possible to find the V3 and to measure its diameter using transcranial B-mode image [49]. However, when the ventricles are compressed, TCCS does help finding the V3 and measuring the MLS. Therefore, we use the term “US” to represent the whole measurement process including arterial flow identification using TCCS in the following sections. To validate the MLS measurement by US, a corresponding CT image within a given time window, usually hours, is used as the gold standard [4, 31, 34, 35]. Because the US scanning plane is approximately horizontal, sonographic MLS and CT MLS measurements were usually compared directly without any transformation or conversion.

Stolz et al. prospectively recruited 61 patients with supratentorial infarction (45 patients) or intracerebral hemorrhage (16 patients) [33]. A total of 122 bedside sonographic measurements of MLS were compared with CT data in a 12-hour time window. The overall correlation coefficient was 0.93. For the 50 US measurements taken within a 3-hour window, the correlation was even better. The overall 95% confidence interval of the MLS difference between TCCS and CT measurements was ±1.78 mm. All differences were less than 2 mm. In addition to validating their results, the authors concluded that US is particularly suitable for critically ill patients who are not fit for transportation. They did not report whether any patient was excluded because of insufficient temporal acoustic window.

After confirming the accuracy of sonographic MLS measurement, these authors enrolled 42 with acute, severe hemispheric stroke defined as having Scandinavian stroke scale scores of less than 35 points [34]. CT and carotid duplex sonography were performed on admission. TCCS was carried out 8 ± 3, 16 ± 3, 24 ± 3, 32 ± 3, and 40 ± 3 hours after stroke onset. Infarction size was determined from follow-up CT. Twelve of their patients died as a result of cerebral herniation and 28 survived. Two men received DC 27 and 30 hours after stroke and survived. They were excluded from further analysis. MLS was significantly higher in the herniation group as early as 16 hours after onset of stroke. The mortality was 100% when sonographic MLS was larger than 2.5, 3.5, 4.0, and 5.0 mm after 16, 24, 32, and 40 hours, respectively. Sixteen of 42 patients were sedated and artificially ventilated during the first 48 hours, making clinical monitoring extremely difficult. The authors suggested that bedside TCCS monitoring of MLS is a diagnostic alternative in critically ill patients, who cannot otherwise be monitored adequately.

Tang et al. evaluated 51 consecutive patients with acute spontaneous supratentorial ICH using US [35]. Eighteen patients were excluded for poor temporal acoustic bone windows at least one side of the skull. In addition to MLS, they also measured the pulsatility index (PI) of the MCA and compared it with CT data, including MLS and hematoma volume calculated using the *ABC* formula. The correlation coefficient between the MLS by US and by CT was 0.91. Compared with ICH volume less than 25 mL, those with greater volume had larger MLS and higher PI of the ipsilateral MCA. By using US, MLS was more sensitive and specific than PI in detecting large ICH and predicting poor outcome. The authors confirmed the accuracy of sonographic MLS measurement and also concluded monitoring MLS by US can detect hematoma expansion and predict short-term functional outcome. They did provide a patient whose hematoma expansion was detected by US and confirmed by follow-up CT, but whether there were other patients having similar courses was not reported.

Llompart Pou et al. prospectively conducted 60 bedside TCCS studies in 41 TBI patients with an average time interval between cranial CT and TCCS studies of 322 ± 216 min [32]. According to Marshall (TCDB) classification, 11 of their 60 CT studies were type V (evacuated mass). However, the authors did not report further details about the surgeries performed. No patient was excluded because of an insufficient acoustic window. The correlation coefficient between the MLS measured by CT and by TCCS was 0.88. Differences between them ranged from +2.33 to −2.07 mm with an average of 0.12 mm. There were no statistically significant differences in any subgroup. The authors arrived at a similar conclusion that sonographic MLS measurement is accurate and suitable for bedside monitoring in TBI patients.

Sonographic MLS measurements using the V3 as a landmark is accurate compared to CT slices at the level of the V3 [4, 31, 34, 35]. However, direct comparison of sonographic MLS data with CT MLS data measured at the SP is inappropriate because the maximal anterior-posterior diameter of the V3 is caudal (inferior) and posterior to the SP. Motuel et al. conducted a prospective study on 52 consecutive neurosurgical intensive care unit patients, and of them 31 were admitted for severe TBI [4]. Seven patients had had surgery to remove intracranial mass. Sonographic MLS was measured as soon as possible before or after CT using the V3 as a landmark. In addition to comparing them to CT MLS data at the V3 (method 1), the authors also compared their sonographic MLS data to “standard” CT MLS data at the SP (method 2). The correlation coefficient was 0.76 for method 1 and 0.81 for method 2. The difference between US and CT measurements averaged 0.1 mm for method 1 and 0.9 mm for method 2.

Although not statistically significant, the authors did report slightly smaller MLS measured by CT using the V3 as a landmark (4.2 ± 5.5 mm) compared to MLS obtained using the SP (4.7 ± 6.7 mm). The relationship between MLS and ICP was studied by examining the results from the 30 patients with invasive ICP monitoring. No significant correlation was found between ICP and MLS as assessed using all three methods. Such results suggested that MLS is not uniform across the subfalcine space and anatomical constraints play a role in determining MLS at different anatomical markers. Similarly, there were also differences between the MLS determined using the SP and the MLS using the pineal gland as measured on CT images, even when they are on the same slice [19, 22, 25]. Based on these results, MLS measurements seem to be comparable only when the same landmark is used.

## 3. Algorithms for Automated Midline Shift Measurement

Computer-aided imaging diagnosis systems have significant potentials for assisting human experts in evaluating brain images. In addition to identifying intracranial lesions, measurement of MLS should be an important component of these systems. In this section, we review algorithms that can measure MLS automatically. Most of them are based on CT images but can be easily modified to work on MRI images.

For a human specialist, measuring the MLS on images of a given study is fairly straightforward. After picking up the right axial slice or level and finding the reference point determined either by the iML or by the midpoint of the width of the intracranial space, MLS can be measured as the perpendicular distance between the landmark (the SP or the pineal gland) and the reference point. It is easy for a computer system to measure distances on digital images. However, specialized preprocessing and feature extraction techniques must be applied to find the pertinent points on the input images before actually measuring the MLS. A number of methods that detect the intact midsagittal plane (iMSP) on a complete brain CT study [50–53] can be used to provide information about the iML on the single slice used to measure the MLS. Moreover, to measure the “standardized” MLS at the level of the FM, the correct slice must be correctly identified manually or automatically.

Algorithms that measure MLS are classified into two types: symmetry-based and landmark-based ones. In symmetry-based algorithms, recognition of specific anatomical landmarks is unnecessary. Instead, a curve connecting all displaced and deformed structures is sought. Since some structures such as the SP and the pineal are displaced by an intracranial mass, while others such as the ventricles and the corpus callosum are deformed, we use the term “deformed midline (dML)” to collectively describe this curve [54]. In landmark-based algorithms, specific structures, often parts of the lateral ventricles, are recognized first. Within the given (ventricular) regions, the SP or another landmark is identified and the MLS is measured accordingly.

### 3.1. Symmetry-Based Methods

Liao et al. proposed an automated method to recognize the dML on CT slices at the level of the FM [54]. As shown in Figure 2(b), the dML was decomposed into three segments: the upper and the lower straight segments (black lines) representing parts of the tough falx cerebri separating two brain hemispheres, and the central curved segment formed by a quadratic Bezier curve (white curve), representing the intervening soft brain tissue. The authors assumed that the dML is the curve with maximal bilateral symmetry, calculated by minimizing the summed square of the differences across all midline pixels over a horizontal (left-right) range of 24 mm. To further simplify the computation, the upper and lower falx segments were assumed to be immobile, turning them into vertical lines. A genetic algorithm was applied to derive the optimal values of the four variables determining positions of the three control points of the Bezier curve. The algorithm was repeated three times with the maximally allowed values of MLS set at 15, 22.5, and 30 mm. If the results were stable, the MLS was readily determined by the position of the central control point after detecting the dML. Otherwise they were considered failures.

Our algorithm was evaluated on pathological images from 81 consecutive patients treated in a single institute over a period of one year. Fifty-four of these patients had TBI and 25 had spontaneous ICH. Our algorithm was able to measure the MLS of 65 (80%) patients. In 62 (95%) of them the difference was less than 1 mm. All three inaccurate results occurred in images with MLS larger than 10 mm. Although the success rate of MLS measurements decreased with increasing MLS, most patients with MLS larger than 5 mm were correctly measured. A major drawback of our algorithm was the higher failure rate in images of spontaneous ICH, which often occurs at basal ganglia near the midline. Using manually and automatically measured MLS data, we also performed outcome analysis in TBI patients [7]. Although not statistically significant, MLS seemed to be a predictor for mortality. Prediction of death using an MLS of 3.5 mm as threshold was 76% sensitive (13/17) and 71% (24/34) specific. For mortality prediction, our automated algorithm performed no worse than manual MLS measurement.

Chen et al. proposed an automatic method to estimate the dML on MRI images in glioma patients [55]. The authors constructed an enhanced Voigt model which predicted the location of the dML on the axial slice bearing maximal tumor diameter using lesion size and location. They used an elastic coefficient and a viscosity coefficient of brain tissue from the literature. A composite local symmetry metric combining local intensity symmetry and local intensity gradient symmetry is proposed to refine the predicted midline within a local window whose size is determined according to a pinhole camera model. Without theoretical proof, the authors tried different values of the modulation factor empirically and the candidate with maximum sum of composite local symmetry was treated as the “predicted” dML in each case. Then, this dML was refined and smoothed according to local symmetry.

The proposed method was validated on 30 MRI data sets from Multimodal Brain Tumor Segmentation challenge in MICCAI 2013 conference. The authors manually picked the axial slice with maximum MLS, while they consider it corresponding to the slice with maximal tumor-to-brain ratio. The MLS on these MRI slices ranged between 0 and 6 mm. Although the delineated dML was not at the level commonly used for “standardized” MLS evaluation and outcome assessment, the author did obtain accurate results. Compared to manually traced dMLs, their method yielded a mean difference of 0.61 ± 0.27 mm and an average maximum difference of 1.89 ± 1.18 mm.

### 3.2. Landmark-Based Methods

Yuh et al. developed a suite of computer algorithms, within the MATLAB 7.0.1 programming environment to evaluate CT for evidence of TBI [56]. The algorithm seemed to detect the skull and the iMSP first but details were not provided. Then, blood and CSF pixels were detected using appropriate CT density thresholds, spatial filtering, and cluster analysis. Once pixels containing blood are identified, they are classified as EDH, SDH, ICH, SAH, or IVH according to their location relative to the skull. In order to calculate the MLS, the symmetry of the cerebrospinal fluid pixels in the lateral ventricles was assessed with respect to the iML determined by the symmetry axis of the skull. The volume of the cluster of basal CSF pixels was calculated to determine the status of the basal cisterns. However, the authors did not report how the CSF pixels were identified as ventricles or cisterns. The software was then applied to a validation sample of more than 200 patients evaluated for suspicion of acute TBI. Automated detection of the presence of at least one radiological sign of acute TBI demonstrated high sensitivity of 98%. The authors did not report quantitative MLS measurement results. They reported a sensitivity of 100% and a specificity of 98% for detecting MLS larger than 5 mm. Since there were only 9 patients with such findings and an additional 4 patients have false positive results, the positive prediction rate of their MLS detection method was only 70%.

Xiao et al. proposed a procedure that can measure MLS by recognizing the SP within the given CT study [57]. All slices of the study were fed into a preprocessing system that recognized the skull, and the iMSP and stripped of all extracranial regions using a combination of filters in a multiresolution approach. Then, the slice containing the FHs and the SP was selected from all ventricular regions by expert rules and a multiresolution binary level set method. The iML was defined as the intersection between the iMSP, calculated using Liu's method [53], and the plane of that slice. Finally, the SP is recognized as an isodense line segment within hypodense FHs using Hough transform, weighted by repeated morphological erosion. The farthest point on the SP as perpendicular from the iML was used to measure the MLS. Usually, it was the most posterior point.

Our system was tested on images from 96 consecutive patients admitted to the neurosurgical intensive care unit [57]. The results are evaluated by human experts. Our algorithm failed to recognize FHs in images of 16 patients, all with large intracranial hematoma (13 SDHs, 1 EDH, and 2 ICHs) with marked brain deformation. In 2 cases with cavum septum pellucidum, where SP has a separation between its two leaflets, our algorithm recognized only one of the two leaflets. In the remaining 78 patients, the mean difference between automatic and manual MLS measurements is 0.23 ± 0.52 mm. Markedly deviated SP was successfully recognized and MLS up to 30 mm was accurately measured. The difference between automatically measured and manually measured MLS was less than 1 mm in 70 of 78 cases and less than 0.5 mm in 60. The error did not increase with the larger MLS. Our method is robust and can be applied in emergency and routine settings. Thirty patients underwent surgery. Their average MLS was much larger than those without surgery (9.2 ± 7.1 versus 1.7 ± 1.3 mm, *p* < 0.001), confirming the usefulness of MLS for guiding immediate surgical intervention.

Chen et al. presented an automated system based on CT images that can estimate the MLS and screen for increased ICP [58]. Their method was based on their previous work of ventricle detection [59]. The CSF pixels were detected using a Gaussian mixture model for each CT slice to classify the pixels into four tissue types: bone or hematoma, gray matter, white matter, and CSF. Using these pixels, the ventricles were detected using size and location criteria. To estimate the MLS, the authors first performed iML estimation based on skull symmetry, falx, and interhemispheric sulcus. Then, segmentation of the ventricles from the CT scan was performed and used as a guide for the identification of the dML through shape matching. The authors considered these processes to mimic the measuring process by physicians and showed promising results in the evaluation.

CT data sets containing 391 slices from 17 TBI patients were tested for iML and dML detection, as well as MLS measurement and ICP estimation. In most slices (over 80%), the errors between the iML estimated by their method framework and the manual annotation were around 2 pixels, or about 1 mm. For the dML, above 80% has less than 2.25 mm difference provided that the quality of the ventricular segmentation is relatively good, defined as a segmentation result allowing manual MLS measurement. In other words, the method also failed when the ventricles could not be identified due to marked brain deformation.

Liu et al. presented another landmark-based method of automatically detecting and quantifying MLS shift on TBI CT images [60]. After discretization of histogram, pixels with the images were classified as skull, hematoma, brain, or CSF. The “middle slice,” probably the slice at the level of the FM, was detected from all images in the given study using a probability map containing the FHs, the V3, and the perimesencephalic cistern. On that slice, the anterior and posterior falx attachments were detected within a given range based on skull thickness. A Gaussian mixture clustering process was used to detect the CSF regions and landmark pixels within them. Multiple candidates of falx candidates were detected using directional single connected chain following edge detection. The spatial relationships between these markers were trained from data from 200 patients. The probability distribution is learnt from training data from the middle slice of 200 patients using a Gaussian mixture model.

The authors tested their method on an experimental data set containing 565 patients with about 12 CT slices per patient. Whether the training data overlaps with the testing data was not reported. More than 100 patients had MLS larger than 5 mm. Their method achieved a maximum distance error of 4.7 ± 5.1 mm. The author concluded that their method outperformed previous methods, especially in the cases of large ICH and missing ventricles.

## 4. Newer Applications: Beyond Aiding Diagnosis and Guiding Treatment

### 4.1. Measurement of Posttreatment Midline Shift

Intracranial lesions diagnosed on CT or other images evolve over time. Their shape and size are also changed by medical or surgical treatment. After these treatments, MLS can still be measured using the same methods described in Section 1.3. Patients undergoing DC have parts of their skull removed, making it difficult to measure the width of the intracranial space. However, the iML can still be identified and used to measure MLS. After successful treatment, the MLS should decrease. We defined the midline return (MLR) as follows: MLR = MLS_after_ − MLS_before_, where MLS_after_ and MLS_before_ denote the MLS measured from posttreatment and that from baseline images, respectively [61]. In addition, we proposed some quantitative imaging parameters for evaluation of decompressive efforts and decompressive effects. The effort of DC, the craniectomy volume, can be estimated using the ABC method [62]. On the other hand, the transcalvarial brain herniation (TCH) volume, corresponding to the treatment effect created by skull removal and expansive duroplasty, is modeled as difference between two spherical caps [63].

Takeuchi et al. retrospectively reviewed preoperative and postoperative CT images of 186 consecutive patients who underwent surgery for TBI and investigated the prognostic factors of new CT findings appearing less than 24 hours after surgery [64]. Although there was no standardized or established rule for the timing of postoperative scan, 139 of 186 patients had CT within 1 hour after surgery, including 138 routine follow-up. A total of 30 new findings on postoperative CT were observed in 29 patients (15.6%), including SDH in 11 patients (10 contralateral, 1 ipsilateral), brain contusions in 11 (9 contralateral, 2 ipsilateral), contralateral EDH in 5, and whole-brain ischemia in 3. The authors did not report postoperative MLS on follow-up CT examinations. Ten patients with new findings underwent a total of 11 subsequent surgeries, and 7 of them had DC. A univariate analysis showed that GCS score of 8 or less, SDH as the primary indication for surgery, MLS, obliterated basal cistern, and DC were significantly associated with higher risk for new findings. Since DC was performed as the first procedure in 26 of 29 patients with new findings, 24 of them having removal of SDH with mass effect including large (9.0 ± 5.7 mm) MLS and basal cistern obliteration, and these factors were indeed closely related. Multiple logistic regression analysis revealed DC, low GCS, and basal cistern obliteration as significant risk factors.

Sucu et al. evaluated 45 patients with cSDH who underwent burr-hole or twist-drill craniostomy [25]. Although MLS was measured both in the preoperative and in the early postoperative CT images, only the preoperative MLS correlated with improvement in 28 patients with impaired consciousness prior to surgery. However, the authors did observe MLS reduction, or MLR, at both SP and pineal gland in most patients. The MLR probably contribute to improvements of symptoms other than consciousness recovery, such as hemiparesis or headache. Measuring postoperative MLS alone probably plays a smaller role in cSDH because clinical improvement can be achieved even with partial evacuation leaving residual cSDH and MLS [47, 65].

Jeon et al. studied 70 patients with malignant MCA infarction who underwent DC [66]. MLS was measured at the SP and pineal gland on the last preoperative and postoperative CT images with an average median interval of 8.3 hours. Reduction in MLS, or MLR, was associated with higher postoperative GCS scores and lower mortality at 6 months after stroke after adjusting for age, sex, NIHSS score, and preoperative MLS. The anterior-posterior diameters of the bone flaps created by DC were approximately 130 mm. The “extracranial bulging volume,” the volume of the brain tissue beyond the surface formed by skull window edge created by DC, was significantly related to MLS reduction. On average, patients with MLS reduction have the smallest infarct volume and those with MLS progression have the largest. However, the difference was not significant. Whether larger DC may lead to larger MLS reduction remains unknown. Instead of measuring the extracranial bulging volume, our geometric model of the TCH may provide a more precise estimate about the decompressive effect [63].

Missori et al. evaluated preoperative and early postoperative CT images of 73 patients with unilateral DC [67]. The early postoperative MLS was measured on images obtained within 3 postoperative days. The reasons for DC were hemorrhagic or ischemic stroke in 48, TBI in 22, and infection in 3. The only factor associated with survival 12 months after surgery was a reduced postoperative MLS at SP from a preoperative average of 9.2 ± 3.8 mm to 2.3 ± 2.7 mm in 42 surviving patients. On the other hand, the MLS reduced less effectively, from 11.5 ± 4.8 mm to 4.7 ± 4.8 mm, in 31 deceased patients. The authors removed relatively small bone flaps, with surface areas of 7643 mm2 in surviving patients and 7372 mm2 in deceased patients. They suggested that some patients should have had a wider DC to increase the probability of survival, probably by further decreasing ICP and reducing the MLS. To help pre- and intraoperative decision making, our formula provides an easy method for estimating the volume of the proposed bone flap, that is, the decompressive effort [62].

In addition to DC, MLS was also used as a neuroanatomical predictor of awakening in acutely comatose patients. Kowalski et al. performed a prospective observational study which included all new onset coma patients admitted to the Neurosciences Critical Care Unit over 12 consecutive months [68]. CT scans were analyzed independently at coma onset, after awakening, and at follow-up. MLS was measured at the SP and pineal gland. Of the 85 patients studied, the mean age was 58 ± 16 years, 51% were female, and 78% had cerebrovascular etiology of coma. The authors did not describe how they treated these patients, either medically or surgically. A total of 43 patients awakened. On CT examined at coma onset, extent of pineal MLS was less pronounced in those patients who awakened. Time elapsed between coma onset CT and follow-up CT was similar for patients who awakened (median 4 days) and those who did not (median 3 days). On follow-up CT, MLS less than 6 mm at SP and pineal gland was associated with coma emergence. Reversal or limitation of lateral brain displacement is associated with acute awakening in comatose patients. The authors suggested MLS can be an objective parameter to guide prognosis and treatment in these patients. Additional independent predictors of awakening were younger age, higher GCS score at coma onset, and nontraumatic coma etiology.

### 4.2. Development of Novel Imaging Features of Mass Effect

Derived from TBI studies, perimesencephalic cisternal compression and MLS are imaging features representing mass effect. By definition, mass effect, which causes increased ICP and impaired cerebral perfusion by itself, is secondary to intracranial mass such as EDH or SDH. Such “secondary injury” is pathophysiologically different from damage inflicted by the intracranial mass, or “primary injury.” Therefore, features of an intracranial mass, such as its volume or thickness, and those of mass effect, are treated as different variables which affect patient outcomes independently and are listed as separate items in a guideline [40]. Mizutani et al. performed multiple regression analysis to investigate the relationship between initial ICP and findings of the first CT scan for 100 consecutive moderate-to-severe TBI patients [69]. They were able to estimate ICP in 80% of patients. Listed in order of importance, CT features that contributed to ICP estimation include cisternal compression, size of SDH, ventricular size, status of SAH, status of cerebral contusion, MLS, and ventricular index. These variables can be grouped into those representing primary injury and those representing secondary injury.

However, Quattrocchi et al. did find an interaction between hematoma size and MLS [14]. When patient outcome and mortality rates are considered, their study indicated that an MLS out of proportion to the thickness of intracranial hemorrhage, measured radially from the inner table of the skull, was a highly useful predictor of poor patient outcome following TBI. Similar interaction was rediscovered by Bartels et al. [70]. They found that MLS in relation to SDH thickness predicted mortality. A total of 59 patients undergoing SDH evacuation and intensive treatment for increased ICP were included, of whom 29 died. They found a strong correlation between an MLS exceeding hematoma thickness by 3 mm or more and subsequent mortality. In these 8 patients, it appeared that the trauma resulted in more damage than just an acute SDH. Similar to large MCA infarcts, this additional damage causes the brain to swell, aggravating the MLS. The authors concluded that the relation between MLS and hematoma thickness could be included as a separate factor for outcome prediction.

Since the MLS is measured at the SP, it is certainly affected by changes in ventricular shapes and sizes. Toth et al. performed a retrospective in 76 adults with severe blunt TBI requiring a ventriculostomy [71]. They quantified left and right lateral ventricular volumes by computer-assisted manual volumetric measurements. Sixty patients had no or small (less than 5 mm) MLS on the initial CT scan. Of these, 15 patients developed MLS larger than 5 mm subsequently. Admission lateral ventricular size ratio (LVR) of more than 1.67 was shown to predict subsequent large MLS with a sensitivity of 73.3% and a specificity of 73.3%. They concluded that LVR analysis is simple and rapidly accomplished and may allow earlier interventions to attenuate later MLS. Whether ventriculostomy would modify their measurement was not discussed.

## 5. Conclusions and Future Directions

Midline shift is a well-proven composite imaging sign that can be measured on CT, MRI, and US. Standardization of MLS measurement facilitates communication and comparison between different raters and permits further automation. We have summarized current state of the art in MLS measurement and its relationship to other clinical and imaging parameters. Characteristics, limitations, and validation of automated algorithms that help measuring MLS were reviewed. We have also highlighted novel imaging parameters or their combinations that may lead to a better understanding of brain displacement and deformation as well as their clinical implications. In addition to refining current practice of MLS measurement on axial CT, MRI, and US images, evaluating MLS on coronal slices or 3-dimentional volumes will provide further information that can be used to optimize medical or surgical treatments of intracranial mass and its mass effect.

## Figures and Tables

**Table 1:** Imaging methods for measuring midline shift and their applications.

Method	Disease or indication	Related references
Computed tomography	Traumatic brain injury	[10–18]
	Middle cerebral artery infarction	[19–21]
	Spontaneous intracerebral hemorrhage	[22, 23]
	Chronic subdural hematoma	[24, 25]
	Brain abscess	[26]
	Cranioplasty	[27]

Magnetic resonance imaging	Middle cerebral artery infarction	[28]
	Cerebral venous thrombosis	[29]^†^
	Brain tumor	[30], [31]^†^
	Brain abscess	[26]

Ultrasound	Traumatic brain injury	[4, 32]
	Middle cerebral artery infarction	[4, 33, 34]
	Spontaneous intracerebral hemorrhage	[4, 33, 35]

Reference number followed by a dagger (†) denotes studies that do not demonstrate significant correlation to other variables.
